# Membrane type 1-matrix metalloproteinase induces epithelial-to-mesenchymal transition in esophageal squamous cell carcinoma: Observations from clinical and *in vitro* analyses

**DOI:** 10.1038/srep22179

**Published:** 2016-02-26

**Authors:** Lijuan Pang, Qiuxiang Li, Shugang Li, Jianwei He, Weiwei Cao, Jiaojiao Lan, Bin Sun, Hong Zou, Chengyan Wang, Ruixue Liu, Cuilei Wei, Yutao Wei, Yan Qi, Jianming Hu, Weihua Liang, Wen Jie Zhang, Mei Wan, Feng Li

**Affiliations:** 1 Department of Pathology and Key Laboratory of Xinjiang Endemic and Ethnic Diseases (Ministry of Education), Shihezi University School of Medicine, Shihezi 832002, Xinjiang, China; 2 Department of Pathology, Beijing ChaoYang Hospital, Capital Medical University, Beijing, 100020, China; 3 Department of Public Health, Medical School, Shihezi University School of Medicine, Shihezi 832002, Xinjiang, China; 4 Department of Clinical Laboratory, First Affiliated Hospital to Shihezi University School of Medicine, Shihezi 832008, Xinjiang, China; 5 Department of Stomatology, First Affiliated Hospital to Shihezi University School of Medicine, Shihezi 832008, Xinjiang, China; 6 Department of Thoracic and Cardiovascular Surgery, First Affiliated Hospital to Shihezi University School of Medicine, Shihezi 832008, Xinjiang, China; 7 Department of Orthopedic Surgery, Johns Hopkins University School of Medicine, Baltimore, MD 21205, USA

## Abstract

Membrane type 1-matrix metalloproteinase (MT1-MMP) is associated with enhanced tumorigenicity in many cancers. A recent study has revealed that MT1-MMP induces epithelial-to-mesenchymal transition (EMT) in prostate and breast cancer cells. However, its role in esophageal squamous cell carcinoma (ESCC) has not been studied. Here, we investigated the role of MT1-MMP in the dissemination of ESCC. Expression of MT1-MMP was detected by immunohistochemistry and tissue microarray in 88 Kazakh ESCC patients. Western blotting was performed to detect endogenous and overexpressed exogenous MT1-MMP in the Eca109 and Eca9706 cell lines, respectively. Transwell assay was used to estimate MT1-MMP-induced invasion and metastasis. EMT-associated proteins were detected by immunohistochemistry and western blotting. The associations between the expression of MT1-MMP and EMT-associated proteins with clinicopathologic parameters were analyzed. Overexpression of MT1-MMP was confirmed in Kazakh ESCC patients. MT1-MMP levels were found to be correlated with the depth of tumor infiltration. MT1-MMP induced EMT in ESCC both *in vivo* and *in vitro*, N-cadherin and Vimentin expression was upregulated upon MT1-MMP transfection into cells. However, E-cadherin was found to be downregulated. MT1-MMP-induced EMT led to increase migration and invasion in ESCC cell lines. In conclusion, our results suggest that MT1-MMP promotes ESCC invasion and metastasis.

Esophageal carcinoma (EC) is a common malignant tumor characterized by its distinct geographic distribution and ethnic distribution^1^. The incidence and mortality rates of EC are high in China. In particular, EC is the leading cause of cancer death, with a mortality rate of 39.27/1,00,000 in the Kazakh ethnic group, in the northwestern part of China^2^. More than 90% of EC cases are esophageal squamous cell carcinomas (ESCC). Clinically, ESCC is characterized by rapid progression and poor prognosis^3^. Moreover, ESCC is associated with a high mortality rate, which is attributed to local invasion and metastasis of the tumor cells at the advanced stage.

Metalloproteinases (MMPs) are Zn^2+^-binding endopeptidases that degrade various components of the extracellular matrix (ECM). MMP levels are elevated in many tumors, and MMPs have been implicated in cellular migration, invasion, and metastasis of tumor cells^4^,^5^,^6^. Several studies have suggested that MMP expression is elevated in ESCC patients^7^,^8^,^9^. Membrane type-1 MMP (MT1-MMP), also known as MMP14, is a membrane-anchored MMP that is widely expressed in tumors. MT1-MMP is frequently associated with enhanced tumorigenicity of many types of cancers^10^,^11^,^12^. The proteolytic activity of MT1-MMP plays a critical role in cancer metastasis^13^. MT1-MMP is involved in the localized proteinase-driven migration of tumor cells through the matrix, leading to secondary lesions. Recently, MT1-MMP was demonstrated to induce epithelial-to-mesenchymal transition (EMT) in prostate^14^ and breast cancer cells^15^,^16^. EMT is a key step in tumor invasion and metastasis, and the induction of EMT leads to the downregulation of E-cadherin, expression of distinct mesenchymal markers such as vimentin, fibronectin, and N-cadherin, and morphological changes^17^. However, very little is known regarding the relationship between MT1-MMP activity and invasiveness of ESCC. In this study, we sought to determine whether MT1-MMP plays an essential role in promoting the invasive behavior of ESCC *in vivo* and *in vitro*. We also investigated whether MT1-MMP-induced EMT plays an important role in the invasiveness of ESCC in order to elucidate the mechanisms underlying MT1-MMP-induced ESCC dissemination.

## Materials

### Patients and cell lines

#### Tissue Samples

Eighty-eight ESCC specimens were collected in this study. The samples were obtained from 88 Kazakh patients who underwent resection of primary carcinoma without receiving radiotherapy or chemotherapy before surgery. The surgeries were performed at the Department of Thoracic Surgery of the First Affiliated Hospital, Shihezi University School of Medicine and the Yili Friendship Hospital, Xinjiang. Forty-two paired-normal esophageal tissue specimens taken from a site distant from the cancerous lesion served as controls. Among the 88 patients, 55 were males and 33 were females. Twenty-five tumors were well-differentiated squamous cell carcinomas, 54 were moderately differentiated, and 9 were poorly differentiated. The diagnosis was confirmed by two senior pathologists. The study was approved by the internal review board of the Shihezi University School of Medicine and the collaborating Yili Friendship Hospital. The protocols were approved by the Ethics Committee of the two Hospitals for tissue sample collection and informed consent was obtained from all subjects. The methods were carried out in accordance with the approved guidelines.

## Methods

### Immunohistochemistry

Immunohistochemical staining was performed on formalin-fixed, paraffin-embedded tissue sections; 5-μm thick sections were used. The biopsy paraffin-embedded tissue array was prepared. MT1-MMP was detected using rabbit monoclonal primary antibody (EPITOMICS) at a dilution of 1:400. Slides were incubated with the following primary antibodies overnight at 4 °C: mouse anti-E-cadherin (1:100; DAKO System), anti-vimentin (1:800; DAKO System), anti-N-cadherin (1:400; Abcam), anti-snail (1:800; Abcam), and anti-slug (1:400; Abcam). The specificity of the immunostaining reaction was verified by referring to previously confirmed positive- and negative-control tissue sections. Immunohistochemical staining was assessed by scoring, as described previously^18^. All patients in this study provided informed consents, and the study was approved by the institutional ethics committee at the First Affiliated Hospital of Shihezi University School of Medicine.

### Cell culture

Human ESCC cell lines Eca109 and Eca9706 were purchased from the Shanghai Institute of Biochemistry and Cell Biology (Shanghai, China). Cells were maintained in Roswell Park Memorial Institute (RPMI) 1640 (HyClone) medium supplemented with 10% fetal bovine serum (GIBCO), 100 U/mL penicillin, and 100 μg/mL streptomycin, in a humidified atmosphere containing 5% CO_2_ at 37 °C.

### Plasmids and cell transfection

Cytosolic MT1-MMP biosensor expression plasmid was constructed by polymerase chain reaction (PCR) amplification of the fused full-length gene encoding the MT1-MMP biosensor (a gift from Dr. Yingxiao Wang at Department of Bioengineering, Institute of Engineering in Medicine, University of California, San Diego, United States) and subcloning into pcDNA3.1 (Invitrogen) between the BamHI and EcoRI sites. Cells (1 × 10^5^) cultured in a 6-well cell-culture plate were transiently transfected with 3.5 μL of MT1-MMP biosensor or empty vector (negative control) using Lipofectamine® 2000 (Invitrogen), according to the manufacturer’s protocol. Transfection efficiency was assessed by monitoring green fluorescent protein expression in Eca109 and Eca9706 cells.

### Cellular immunohistochemical and immunofluorescent staining

Eca109 and Eca9706 cells were transiently transfected with the MT1-MMP construct and seeded onto coverslips. After 24 h, cells were fixed with 4% paraformaldehyde. For immunofluorescence, Eca109 and Eca9706 cells seeded on gelatin-coated coverslips were stained with anti-MT1-MMP antibody and incubated with the secondary antibody (goat anti–rabbit immunoglobulin G fluorescein isothiocyanate; Cell Signaling). Samples were examined with an Axiovert 200M fluorescence microscope (Zeiss, Jena, Germany). Images were acquired with a 40/1.00 objective linked to an AxioCam HR camera. For immunocytochemistry, cells were stained with anti-MT1-MMP antibody and incubated with the secondary antibody (Mouse anti–rabbit; DAKO System).

### Western blot

Cellular proteins were prepared using cell lysis buffer (50 mM Tris-HCl, pH 8.0, 1% NP-40, 2 mM ethylenediaminetetraacetic acid, 10 mM NaCl, 2 mg/mL aprotinin, 5 mg/mL leupeptin, 2 mg/mL pepstatin A, 1 mM dithiothreitol, 0.1% sodium dodecyl sulfate, and 1 mM phenylmethylsulfonyl fluoride). Equal amounts of protein (50 μg) were separated by 10% sodium dodecyl sulfate-polyacrylamide gel electrophoresis and then transferred to nitrocellulose membranes (NY, USA) by electroblotting. The membranes were blocked with 5% bovine serum albumin in Tris-buffered saline and Tween® 20 (10 mM Tris-HCl, pH 8.0, 150 mM NaCl, and 0.05% Tween® 20) for 1 h, and then incubated with rabbit anti-human MT1-MMP antibody (1:1,000; EPITOMICS) overnight at 4 °C before subsequent incubation with horseradish peroxidase-conjugated goat anti-rabbit antibody (BD) for 1 h at 37 °C. The expression level of MT1-MMP protein was analyzed using the LabWork 4.0 program (UVP) and was normalized to that of β-actin. In order to detect EMT proteins, the blots were incubated with the following primary antibodies overnight at 4 °C: rabbit anti-E-cadherin (1:500; Abcam), mouse anti-vimentin (1:100; Santa Cruz Biotechnology), and anti-N-cadherin antibodies (1:1,000; Abcam). This procedure was followed by incubation with sheep anti-mouse (1:20,000) or anti-rabbit (1:20,000) horseradish peroxidase-labeled secondary antibodies for 2 h at room temperature. The bands were detected with an ECL reagent kit (Thermo Systems). β-actin immunoblots served as loading controls.

### Cell migration

A wound healing assay was performed to test cell migration. Cells were grown to 90% confluence and subdivided into a 6-well cell culture plate precoated overnight with purified human laminin-5 (1 g/well) at 4 °C. After transient transfection with the MT1-MMP construct or control plasmid, the cells were incubated for 12–16 h in a humidified incubator at 37 °C and 5% CO_2_. An injury line was made using a 2-mm-wide plastic pipette tip. After incubation, the excess liquid in the wells was removed, and the wells were rinsed with phosphate-buffered saline and covered with serum-free medium. The cells were incubated for 48 h to allow the cells to grow. The cell migration area was quantified by analyzing images, and photographs were acquired at different time points.

### *In vitro* invasion assays

MT1-MMP and pcDNA3.1 control vectors were transiently transfected into ESCC cells (5 × 10^4^), which were then allowed to migrate for 24 h through Transwell® cell culture inserts (8-μm pore size, 6.5-mm diameter; Costar, Cambridge, MA). Transwell® inserts (8-μm pore size; BD Falcon) were coated with Matrigel® toward the lower compartment and filled with 600 μL of Dulbecco’s Modified Eagle’s Medium supplemented with 20% fetal bovine serum. After incubation at 37 °C in a humid atmosphere for 24 h, filters were rinsed with phosphate-buffered saline, fixed with 4% paraformaldehyde (15 min at -4 °C), and stained with crystal violet (0.1%; Thermo Fisher Scientific) for 15 min. Cells on the upper surface of the filters were removed with a cotton swab. Cells that migrated through the filters were counted under the microscope at a magnification of ×400. Each clone was tested in triplicate in at least in two independent assays. Data were expressed as mean ± standard error of the mean of the average number of cells obtained in each filter.

### Statistical analysis

Statistical analyses of the data were performed with SPSS (version 17.0) software. The statistical association between protein expression and various clinicopathological parameters was determined using the chi-squared test. The differences in means, positive rates, and positive staining intensities were analyzed by Student’s t-test, chi-square test, and Mann-Whitney U test, respectively. The correlations between the expressions of various proteins (MT1-MMP, E-CAD, N-CAD, Snail, and Slug) were analyzed by Spearman correlation. *P*-values less than 0.05 were considered to indicate statistical significance.

## Results

### Immunohistochemical analysis of MT1-MMP protein in Kazakh ESCC and normal esophageal tissues

**Figure 1:**
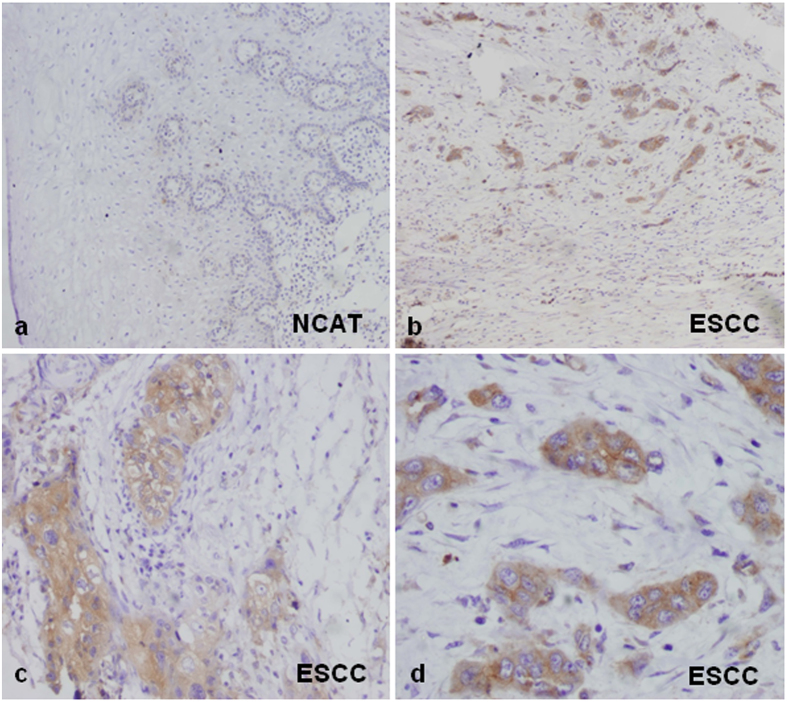
Expression of membrane type 1-matrix metalloproteinase 1 (MT1-MMP) in esophageal squamous cell carcinoma (ESCC) and non-cancerous adjacent tissues (NCAT). (Panel **a**) No detectable expression of MT1-MMP protein in the normal epithelium (100×). (Panel 1**b**) MT1-MMP immunoreactivity was in the invasion front and was increased expression in tumor buds (100×). (Panels **c,d**) Intense cytoplasmic and membranous staining in ESCC (200× and 400×, respectively).

Immunohistochemical analysis for MT1-MMP expression was carried out in 88 Kazakh ESCC specimens and 42 paired-normal esophageal tissue specimens. The results are summarized in Table 1. MT1-MMP expression was observed in 95% (84/88) of Kazakh ESCC specimens, which is significantly higher than that in noncancerous tissues of adjacent normal control esophageal epithelium(Fig. 1a,b). MT1-MMP immunoreactivity was mainly localized in the cytoplasm and plasma membrane of the tumor cells in ESCCs (Fig. 1c,d). Enhanced staining was observed in nests of ESCC infiltrating the stroma, while the nests of surrounding stroma cells exhibited negative expression. MT1-MMP highly expression was in the invasion front and was increased expression in tumor buds (Fig. 1b–d).

The expression level of MT1-MMP in TNM stages III and IV was significantly higher than that in I and II (*P* = 0.008; Table 2). High MT1-MMP levels were associated with invasive lesions. Other clinicopathological factors such as age, gender, tumor location, and tumor differentiation showed no significant correlation with the intensity of MT1-MMP staining (Additional file 1: Table S1).

### MT1-MMP induced morphological changes in ESCC cells

**Figure 2:**
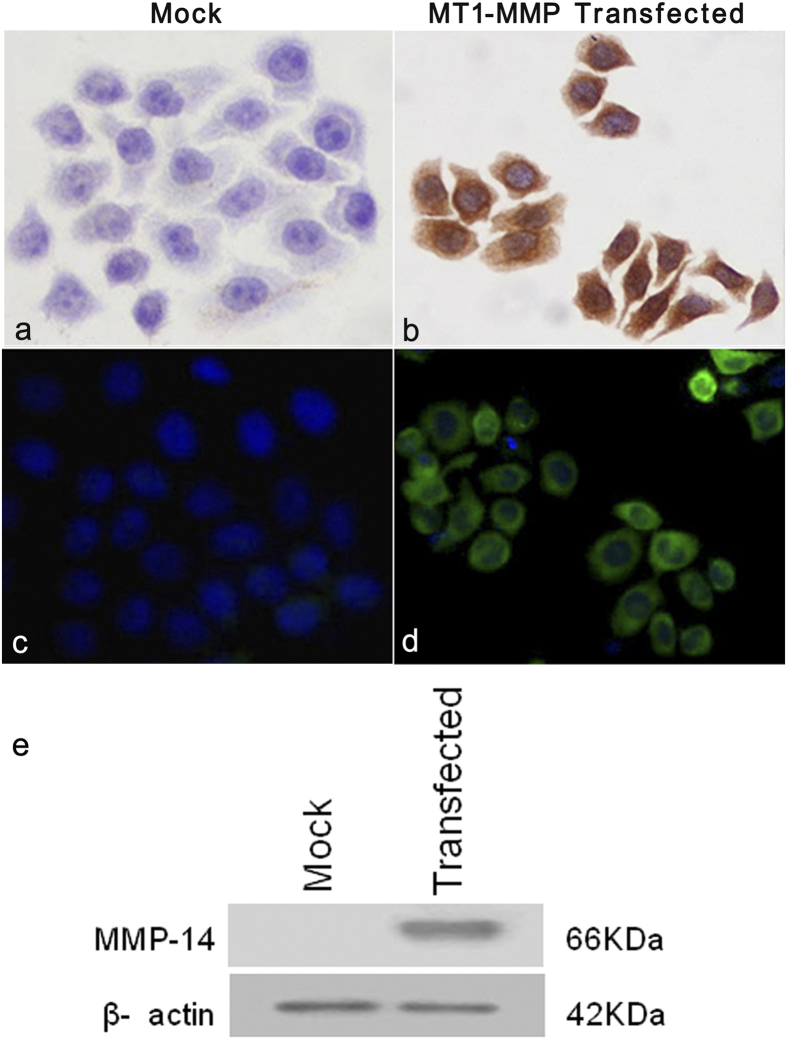
Immunocytochemical and immunocytofluorescent staining, WB of MT1-MMP protein expression in transfected cells. (Panel **a**) Undetectable expression of MT1-MMP protein in ESCC cell lines transfected with pcDNA3.1 vector (Mock; 400×). (Panel **b**) Intense cytoplasmic and membranous staining of MT1-MMP in ESCC cell lines transfected with MT1-MMP plasmids (400×). (Panel **c**) Immunofluorescence image showing the absence of green fluorescence in the cytoplasm and plasma membrane of cell lines transfected with the empty vector (mock; 400×). (Panel **d**) Immunofluorescence image showing green fluorescence in the cytoplasm and plasma membrane of ESCC cell lines transfected with MT1-MMP plasmids (400×). (Panel **e**) Expression of the 42-kDa β-actin protein in cells transfected with the empty vector (mock) and ESCC cell lines transfected with MT1-MMP plasmids by western blotting. The 66-kDa MT1-MMP protein was detected in ESCC cell lines transfected with the MT1-MMP plasmids, but was undetectable in those transfected with mock.

Next, we performed *in-vitro* cell culture studies to examine the role of MT1-MMP in the behavior of ESCC cells. MT1-MMP plasmid was transfected into human ESCC cell lines, and MT1-MMP protein expression was estimated by immunocytochemical and western blot analyses. MT1-MMP overexpression in ESCC cells by transient transfection. Strong exogenous MT1-MMP immunoreactivity was detected in the ESCC cell line with MT1-MMP transfected cells. Control cells transfected with pcDNA3.1 vector did not exhibit detectable immunostaining for MT1-MMP (Fig. 2a). For the MT1-MMP transfected cells, the positive signal was localized in the cytoplasm and plasma membrane of the tumor cells (Fig. 2b). Notably, after transfection with MT1-MMP, the morphology of tumor cells was elongated and fibroblast-like, with decreased cell-to-cell contact (Fig. 2b). Consistent results were achieved in the immunofluorescence assay, in which green fluorescence was observed in the cytoplasm and plasma membrane of tumor cells (Fig. 2d). While no positive signal was detected in control cells by immunofluorescence staining (Fig. 2c). Western blot analysis revealed a 66-kDa MT1-MMP band in transfected ESCC cell lines, but not in control ESCC cell lines (Fig. 2e). However, the 42-kDa β-actin band was detected in both groups of cells.

### ESCC with MT1-MMP expression exhibited increased cell migration *in vitro*

**Figure 3:**
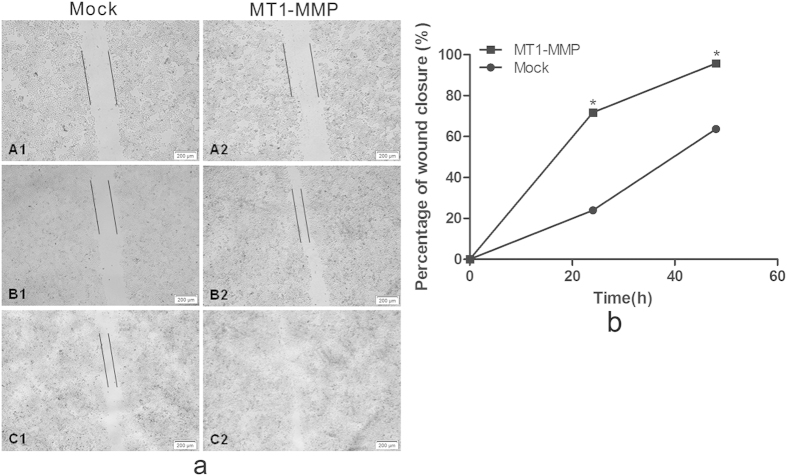
Migration of ESCC cells after MT1-MMP transfection as detected by the wound-healing assay (40×). (Panels **A2–C2**) wound-healing assay of ESCC cells transfected with MT1-MMP plasmid. (Panels **A1–C1**) ESCC cells transfected with control *P < 0.05.

Next, we performed a wound healing migration assay to examine whether MT1-MMP is involved in cell migration. Eca109 cells were transiently transfected with MT1-MMP or empty vector. After 24 h and 48 h, Eca109 cells transfected with MT1-MMP exhibited accelerated cell migration into the wounded area, MT1-MMP overexpressing cells demonstrated a significantly lower percentage of remaining gap in comparison to control cells, whereas wound closure of control cells still showed larger remaining gap (*P* < 0.05, Fig. 3a,b). Thus, MT1-MMP enhanced the cell migration potential of ESCC *in vitro*.

### ESCC with MT1-MMP exhibited accelerated invasion *in vitro*

**Figure 4:**
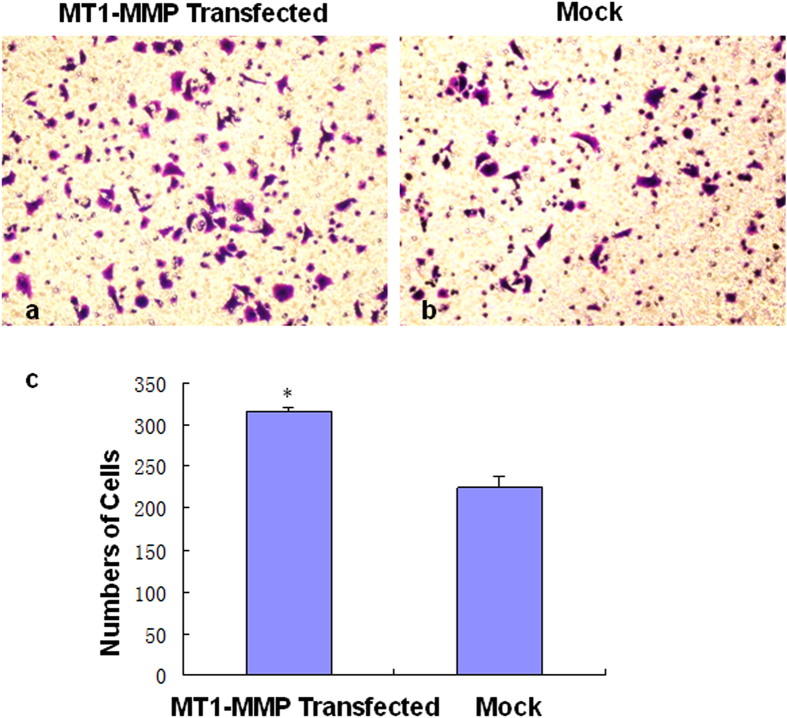
Invasion of Eca109 cancer cells after MT1-MMP plasmid transfection as detected by the Transwell® assay (100×). (Panel **a**) Transwell® assay of Eca109 cancer cells stably transfected with the MT1-MMP plasmid. Increased cell migration was observed in Eca109 cells with ectopic expression of MT1-MMP (*P* < 0.01). (Panel **b**) Transwell® assay of Eca109 cancer cells transfected with the empty vector (mock); (Panel **c**) Quantitative data showing accelerated invasion of ESCC cells with MTP1-MMP overexpression. Number of cells traversing the filters from triplicate determinations was counted; bars, standard error of the mean (SEM). *P < 0.01.

We examined the effect of ectopic expression of MT1-MMP on the invasiveness of ESCC cells. Eca109 cells were transfected with MT1-MMP or empty vector to investigate their ability to migrate through Matrigel®-coated filters for 24 h. The number of MT1-MMP-transfected cells that traversed the pored filters was 317.33 ± 6.43, compared with 225.67 ± 22.37 for the control cells (*P* < 0.05, Fig. 4), indicating that Eca109 cells overexpressing MT1-MMP are more invasive than empty-vector-transfected tumor cells.

### Eca109 and Eca9706 cells with MT1-MMP overexpression underwent EMT

**Figure 5:**
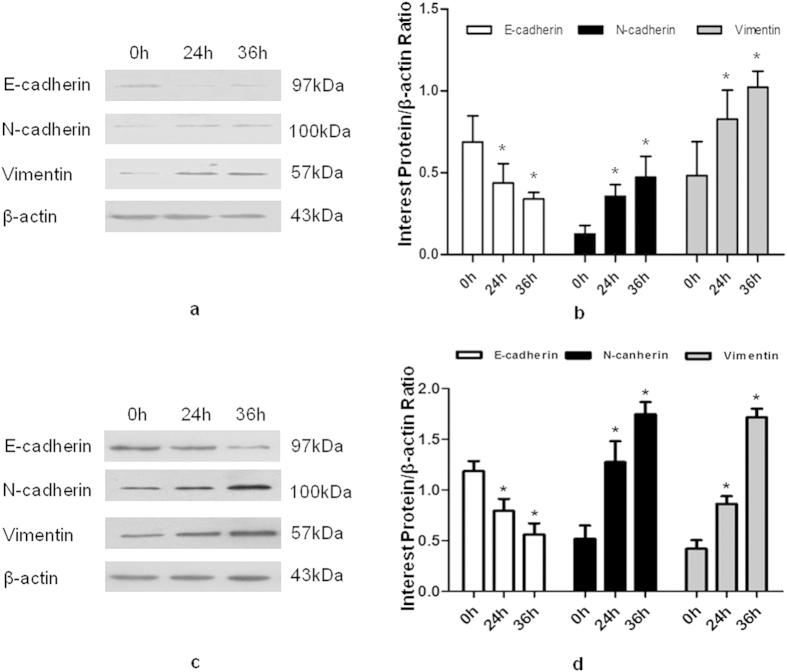
Epithelial (E-cadherin) and mesenchymal markers (N-cadherin, vimentin) were detected by western blot analysis in Eca109 and Eca9706 cells overexpressing MT1-MMP at different time points. (Panels **a,b**) Western blotting and quantitative data showing the expression of E-cadherin, N-cadherin, vimentin, and β-actin in Eca109 cells transfected with MT1-MMP (0, 24, and 36 h). (Panels **c,d**) Western blotting and quantitative data showing the expression of E-cadherin, N-cadherin, vimentin, and β-actin in Eca9706 cells transfected with MT1-MMP (0, 24, and 36 h).

MT1-MMP was transfected into Eca109 and Eca9706 cells for 0, 24, and 36 h. Western blot analysis was performed to detect changes in key markers of EMT. Cells transfected with MT1-MMP exhibited decreased expression levels of the epithelial marker E-cadherin, but increased expression levels of mesenchymal markers N-cadherin and vimentin in Eca109 and Eca9706 cells at 24 and 36 h after transfection (Fig. 5).

### Expression of E-cadherin, N-cadherin, and vimentin in Kazakh ESCC specimens and their correlations with clinicopathological features

**Figure 6:**
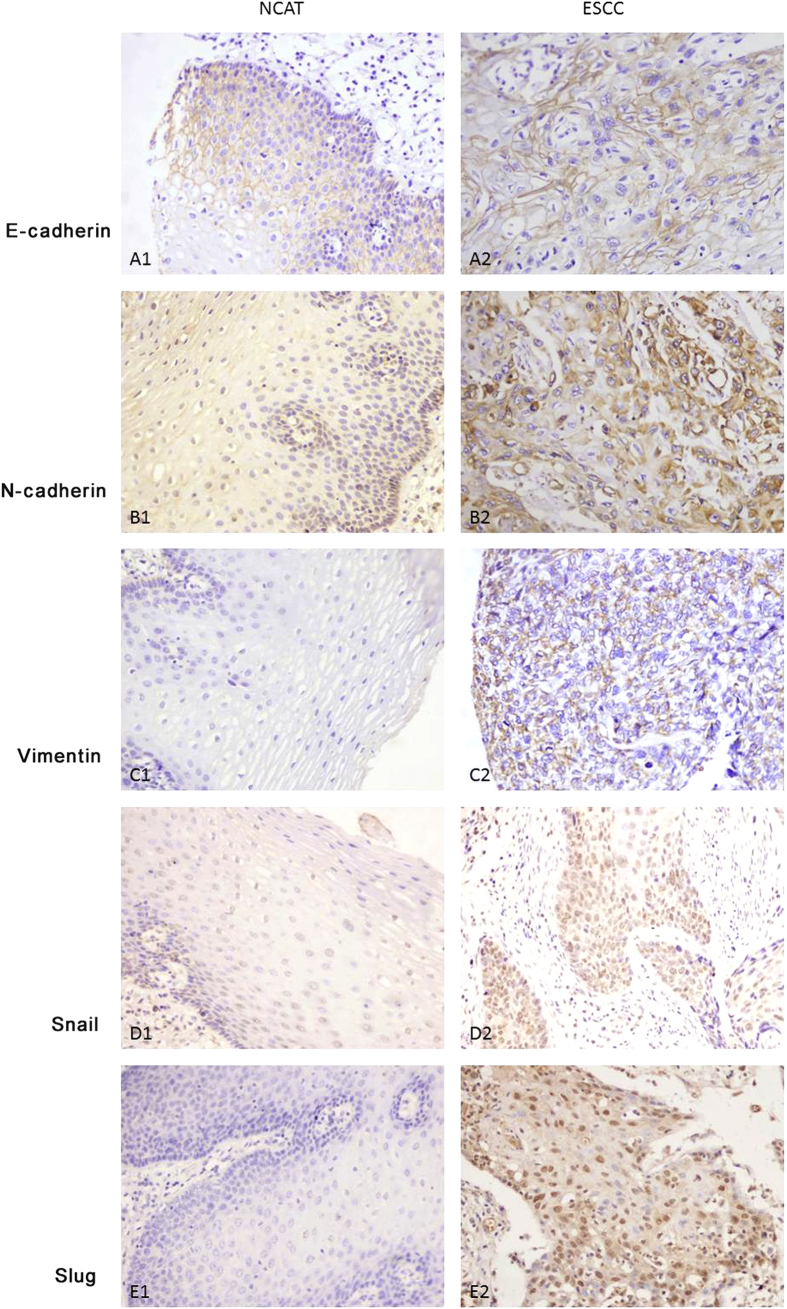
Representative images of immunohistochemical staining for E-cadherin (Panels **A1,A2**), N-cadherin (**B1,B2**), Vimentin (**C1,C2**), Snail (**D1,D2**), and Slug (**E1,E2**) in ESCC and NCAT tissues (200×).

We also examined the expression levels of EMT markers in ESCC patient samples. E-cadherin expression was observed in the membrane of cancer cells in 26.1% (23/88) of tumor tissues (Fig. 6A1) and in 47.6% (20/42) of noncancerous tissues; the difference between these two groups was statistically significant (*P* < 0.05, Table 3). E-cadherin expression was correlated with the following clinicopathologic parameters: tumor differentiation and TNM stage, (*P* < 0. 05, Table 3), but not with sex, depth of invasion, and LN metastasis (*P* > 0.05, Table 3).

Positive expression of N-cadherin was mainly observed in the cytoplasm (Fig. 6B1). Positive expression of N-cadherin was observed in 90.9% (80/88) of tumor tissues, but only in 45.2% (19/42) of noncancerous tissues; this difference was statistically significant (*P* < 0.05, Table 3). Moreover, N-cadherin expression was correlated with cell differentiation *(P* < 0.05), but not with other clinicopathological characteristics (*P* > 0.05, Table 3).

Vimentin was expressed in the cytoplasm of ESCC samples (Fig. 6C1). Positive expression of vimentin was observed in 79.5% (70/88) of tumor tissues, but only in 42.9% (18/42) of noncancerous tissues; this difference was statistically significant (*P* < 0.001, Table 3). Vimentin expression was correlated with depth of tumor invasion, but not with the other clinicopathological characteristics (*P* > 0.05, Table 3).

### Immunohistochemical analysis of Snail and Slug proteins in Kazakh esophageal squamous cell carcinoma and normal esophageal tissues

The zinc finger proteins, Snail and Slug, are important mediators and transcription factors of EMT in tumor cells. Snail and Slug function as E-cadherin repressors and induce EMT. Snail can trigger EMT by repressing the expression of epithelial markers and inducing the expression of mesenchymal markers. This critical step is required for EMT. Therefore, we examined the expression of Snail and Slug in ESCC patient samples.

Snail was expressed in the nucleus of ESCC samples (Fig. 6D1). Positive expression of Snail was observed in 75/88 (85.2%) tumor specimens and 20/42 (47.6%) adjacent tissues; this difference was statistically significant (*P* < 0.001). Furthermore, the presence of Snail was associated with tumor differentiation, depth of tumor invasion and TNM stage (*P* < 0.05, Additional file 2: Table S2). Slug was strongly expressed in the nuclei of ESCC samples (Fig. 6E1). Positive expression of Slug was observed in 65/88 (73.9%) tumor specimens and 18/42 (42.9%) adjacent tissues; this difference was statistically significant (*P* < 0.001). The expression of Slug was associated with the TNM stage (*P* < 0.05, Additional file 2: Table S2). There was no correlation between Slug expression and other clinicopathological characteristics (*P* > 0.05, Additional file 2: Table S2).

### Correlation between MT1-MMP and EMT-associated proteins in Kazakh ESCC specimens

Expression of MT1-MMP in Kazakh ESCC cells was negatively correlated with E-cadherin expression (r = −0.307, *P* < 0.05, Table 4), and positively correlated with N-cadherin and vimentin expression (r = 0.256, *P* = 0.013 and r = 0.593, *P* < 0.001, respectively; Table 4). Transcription factors Slug and Snail were strongly expressed in Kazakh ESCC specimens, and their expression was significantly correlated with reduced E-cadherin expression (r = −0.648, *P* < 0.001 and r = −0.594, *P* < 0.001, respectively; Additional file 3: Table S3). Taken together, the data suggest that MT1-MMP plays an important role in the regulation of EMT in ESCC.

## Discussion

ESCC is an aggressive carcinoma of the gastrointestinal tract with poor prognosis. China has a high incidence of EC^19^, and the incidence of EC in people of Kazakh ethnicity is higher than in other ethnic groups in Xinjiang^20^. The mortality rate for EC among Kazakhs is 55.9/1,00,000, which is higher than the average Chinese rate of 15.2/1,00,000^21^. In recent years, multi-treatment approaches such as surgery, chemotherapy, and radiotherapy have been applied to treat ESCC patients. However, the 5-year survival rate remains poor (approximately 20%). The poor prognosis has been attributed to the invasive and metastatic nature of this tumor. Therefore, understanding the pathogenesis of ESCC and elucidating the key molecules or proteins involved in the process of tumor aggressiveness is particularly important.

MMPs are a prominent family of proteinases associated with tumorigenesis^22^. MMPs play a crucial role in degrading the ECM, leading to cancer cell invasion and metastasis. Twenty-three MMPs have been identified to date, and MMP-1, MMP-2, MMP-3, MMP-7, MMP-9, and MMP-10 are related to esophageal carcinogenesis^23^,^24^. MMP-1 expression in EC cells is associated with poor prognosis; MMP-2 and MMP-3 expression is positively correlated with depth of invasion, lymph node metastasis, and vessel permeation^25^. Elevated expression of MMP-7 and 9 is correlated with invasion depth^26^.

MT1-MMP is a member the membrane-anchored matrix MMP family and is responsible for tissue-remodeling and tumor invasion^26^. Elevated levels of MT1-MMP have been detected in colorectal and breast cancers^27^,^28^. In the present study, we demonstrated that the expression of MT1-MMP increases progressively in EC. MT1-MMP staining was enhanced in nests of ESCC infiltrating the stroma. MT1-MMP expression was significantly lower in normal esophageal epithelium than in tumors. Immunohistochemical analysis revealed that the expression level of MT1-MMP is correlated with clinic stage. In culture, ESCC cells transfected with MT1-MMP exhibited both morphologic and functional changes. The cells lost their epithelial morphology and acquired mesenchymal traits such as fibroblast-like appearance with elongated shape. Wound healing assay revealed that MT1-MMP overexpression led to increased tumor cell migration. Moreover, enhanced invasion and metastasis abilities of the cells were observed with MT1-MMP overexpression in the transwell invasion assay. Our results suggest that the activation of MT1-MMP is an early event in cancer progression and may serve as a marker for invasiveness in esophageal carcinogenesis. Over-expression of MT1-MMP plays an important role in the progression to EC. Yamashita *et al.*^29^ reported that high expression level of MT1-MMP is correlated with poor prognosis in ESCC, and patients exhibiting increased MT1-MMP expression showed recurrence of distant metastasis, indicating that MT1-MMP plays a critical role in ESCC progression. Our results revealed that the expression of MT1-MMP is significantly higher in TNM stages III and IV than in I and II in ESCC patients.

EMT has been identified as a key step in tumor invasion and metastasis. EMT enables cells to change their normal architecture and invade the surrounding environment^30^,^31^,^32^. EMT is associated with loss of epithelial markers and upregulation of mesenchymal markers, and is characterized by morphological changes from typical epithelial phenotype to elongated, spindle phenotype characteristic of fibroblasts, rendering the tumor cells more invasive. Transforming growth factor-β1 has been widely regarded as the key growth factor involved in driving EMT, while MT1-MMP was only recently identified as an inducer of EMT in cancer cells such as prostate cancer^14^,^33^ and oral carcinoma SCC9 cells^17^. In this study, we demonstrated that overexpression of MT1-MMP can lead to EMT in Eca109 and Eca9706 cells. To the best of our knowledge, this is the first study to demonstrate the role of MT1-MMP inducing EMT in ESCC progression. MT1-MMP can induce EMT via transcriptional repression of E-cadherin. When ESCC cells were transfected with MT1-MMP, cell morphology changed from cubic epithelial phenotype to spindle fibroblast-like phenotype. Moreover, decreased expression of epithelial markers (E-cadherin) and increased expression of mesenchymal markers (vimentin and N-cadherin) were observed. Many studies showed both immunochemistry and western blotting MT1-MMP was highly expressed in ESCC. Together, these results suggest that MT1-MMP promotes cancer cell invasion in ESCC through inducing EMT.

Our results also revealed that, in Kazakh-ethnic ESCC, the expression of EMT-associated proteins (E-cadherin, N-cadherin, and vimentin) is significantly associated with lymph node metastasis and distant metastasis. In ESCC tissue samples from Kazakh patients, the expression of E-cadherin was downregulated, while the expressions of N-cadherin and vimentin were upregulated. Importantly, E-cadherin and N-cadherin expression was related to histological differentiation, providing immunohistochemical evidence of EMT. MT1-MMP level was negatively correlated with that of E-cadherin, but positively correlated with those of N-cadherin and vimentin. Snail and Slug are two key factors involved in the transcriptional repression of E-cadherin. Snail and Slug bind directly to E-box motifs on target gene promoters to downregulate E-cadherin expression, and Snail has been proposed to function as a strong repressor of E-cadherin transcription^34^. In the present study, we also investigated the involvement of Snail transcription factor in the regulation of E-cadherin. Positive expression of Snail was significantly associated with reduced E-cadherin expression. This result is consistent with the findings of Rowe, R. G. *et al.* who demonstrated that Snail-mediated increase in MT1-MMP (MMP-14) in fibroblasts promotes growth and invasion in the collagen-rich tumor microenvironment^35^.

In conclusion, our *in vitro* and *in vivo* results suggest that MT1-MMP prompts ESCC invasion and metastasis by repressing E-cadherin and subsequently inducing EMT. Since MT1-MMP is critical for ESCC progression. Our results suggest that the mechanisms of MT1-MMP participating the tumor development and progression not only correlate with direct participating in degradation extracellular matrix but also in inducing EMT by enhancing N-cadherin. Some studies have demonstrated MT1-MMP is an independent poor prognostic factor for ESCC^29^,^36^,^37^. We hope MT1-MMP is becoming potential candidate for promising therapeutic marker in ESCC treatment and judging prognosis in the near future.

## Additional Information

**How to cite this article**: Pang, L. *et al.* Membrane type 1-matrix metalloproteinase induces epithelial-to-mesenchymal transition in esophageal squamous cell carcinoma: Observations from clinical and *in vitro* analyses. *Sci. Rep.* **6**, 22179; doi: 10.1038/srep22179 (2016).

## Supplementary Material

Supplement Table S1

Supplement Table S2

Supplement Table S3

## Figures and Tables

**Table 1:** Expression of MT1-MMP proteins in Kazakh ESCC and control normal esophageal tissue samples.

Groups	Total cases	MT1-MMP expression				U	P
		−	+	++	+++		
ESCC	88	4	44	29	11	**−**4.902	**<0.001**
Control	42	15	19	5	3		

P-values ≤ 0.05 are indicated in bold.

**Table 2:** MT1-MMP protein expression in different TNM stages of Xinjiang Kazakh ESCC patients.

TNM	Cases	−	+	++/+++	U	P
I+II	64	2	26	36	518.000	**0.008**
III+IV	24	2	16	6		

P-values ≤ 0.05 are indicated in bold.

**Table 3:** EMT protein expression in relation to clinicopathological characteristics in Xinjiang Kazakh ESCC.

Clinicopathologica features	N	E-cadherin	*χ*^2^	N-cadherin	*χ*^2^	vimentin	*χ*^2^
		Negative		Negative		Negative	
		Positive	P	Positive	P	Positive	P
Normal	42	22	5.927	23	32.656	24	17.498
		20	**0.015**	19	**0.001**	18	**<0.001**
ESCC	88	65		8		18	
		23		80		70	
Male	55	42	0.475	4	0.587	10	0.466
		13	0.491	51	0.444	45	0.495
Female	33	23		4		8	
		10		29		25	
Age (y)							
<60	41	31	0.121	3	0.292	8	0.042
		10	0.728	38	0.589	33	0.838
≥60	47	34		5		10	
		13		42		37	
Tumor invasion							
Superficial layer	49	40	0.679	5	0.166	5	7.140
		9	0.410	44	0.684	44	**0.008**
Deep layer	39	29		3		13	
		10		36		26	
LN metastases							
Yes	57	15	2.795	4	0.842	9	2.164
		12	0.095	53	0.359	48	0.141
No	31	18		4		9	
		11		27		22	
Tumor differentiation							
Well	25	14	5.772	5	5.029	8	2.861
		11	**0.016**	20	**0.025**	17	0.091
Moderate-poor	63	51		3		10	
		12		60		53	
Stage							
I + II	64	53	9.734	5	0.464	12	0.419
		11	**0.002**	59	0.496	52	0.517
III+IV	24	12		3		6	
		12		21		18	

P-values ≤ 0.05 are indicated in bold.

**Table 4:** Correlation between the expression of MT1-MMP protein and E-CAD, N-CAD, Vimentin, Snail, Slug proteins in ESCC.

MT1-MMP	E-CAD			N-CAD			Vimentin			Snail			Slug		
	−	+	2+/3+	−	+	2+/3+	−	+	2+/3+	−	+	2+/3+	−	+	2+/3+
−	0	0	4	2	1	1	4	0	0	4	0	0	4	0	0
+	7	36	1	4	29	16	9	32	3	6	28	10	11	17	16
2+/3+	16	20	4	2	16	22	5	7	28	3	12	25	8	7	25
	r = −0.30 **P** = **0.004**			r = 0.256 P = 0.013			r = 0.593 **P** < **0.001**			r = 0.460 **P** < **0.001**			r = 0.306 **P** = **0.004**		

P-values ≤ 0.05 are indicated in bold.
