# Supplementary material for: Membrane type 1-matrix metalloproteinase induces epithelial-to-mesenchymal transition in esophageal squamous cell carcinoma: Observations from clinical and in vitro analyses
Source: Sci Rep. 2016 Feb 26;6:22179. doi: 10.1038/srep22179 (PMC4768157; doi:10.1038/srep22179)
Supplement: Supplement Table S1 [file srep22179-s1.pdf]

Membrane type 1-matrix metalloproteinase induces epithelial-to-mesenchymal transition in esophageal squamous cell carcinoma: Observations from clinical and *in vitro* analyses

Lijuan Pang[1#], Qiuxiang Li[1], Shugang Li[2], Jianwei He[3], Weiwei Cao[1], Jiaojiao Lan[1], Bin Sun[4], Hong Zou[1], Chengyan Wang[1], Ruixue Liu[1], Cuilei Wei[1], Yutao Wei[5], Yan Qi[1], Jianming Hu[1], Weihua Liang[1], Wen Jie Zhang[1], Mei Wan[6], Feng Li[1*]

[1]Department of Pathology and Key Laboratory of Xinjiang Endemic and Ethnic Diseases (Ministry of Education), Shihezi University School of Medicine, Shihezi 832002, Xinjiang, China
[2]Department of Public Health, Medical School, Shihezi University School of Medicine, Shihezi 832002, Xinjiang, China
[3]Department of Clinical Laboratory, First Affiliated Hospital to Shihezi University School of Medicine, Shihezi 832008, Xinjiang, China
[4]Department of Stomatology, First Affiliated Hospital to Shihezi University School of Medicine, Shihezi 832008, Xinjiang, China
[5]Department of Thoracic and Cardiovascular Surgery, First Affiliated Hospital to Shihezi University School of Medicine, Shihezi 832008, Xinjiang, China.
[6]Department of Orthopedic Surgery, Johns Hopkins University School of Medicine, Baltimore, MD 21205, USA

# First author: Dr. Lijuan Pang, M.D, Ph.D E-mail: ocean123456@163.com

[*]**Corresponding author: Dr. Feng Li, M.D, Ph.D** Department of Pathology and Key Laboratory of Xinjiang Endemic and Ethnic Diseases (Ministry of Education), Shihezi University School of Medicine (E-mail: lifeng7855@126.com)

Supplement Table S1. Relationship between MT1-MMP protein expression and the clinical, pathological features in xinjiang Kazakh ESCC.

Table S1

Parameters	cases	MT1-MMP expression		p
		-	+	
Gender				
male	55	2	53	0.629
female	33	2	31	
Age (years)				
<60	41	1	40	0.62
≧60	47	3	44	
Histopathological Grading	25	1	24	0.55
Well	54	2	52	
Moderate	9	1	8	
poor				
Lymph node metastasis	31	2	29	0.611
Negative (No)	57	2	55	
Positive (N1)				
Fibrous membranes				
Absent	49	3	46	0.626
Present	39	1	38	
Stage				
I+IIA	29	0	29	0.371
IIB	35	2	33	
III+Ⅳ	24	2	22	
