# Supplementary material for: Membrane type 1-matrix metalloproteinase induces epithelial-to-mesenchymal transition in esophageal squamous cell carcinoma: Observations from clinical and in vitro analyses
Source: Sci Rep. 2016 Feb 26;6:22179. doi: 10.1038/srep22179 (PMC4768157; doi:10.1038/srep22179)
Supplement: Supplement Table S2 [file srep22179-s2.pdf]

Membrane type 1-matrix metalloproteinase induces epithelial-to-mesenchymal transition in esophageal squamous cell carcinoma: Observations from clinical and *in vitro* analyses

Lijuan Pang[1#], Qiuxiang Li[1], Shugang Li[2], Jianwei He[3], Weiwei Cao[1], Jiaojiao Lan[1], Bin Sun[4], Hong Zou[1], Chengyan Wang[1], Ruixue Liu[1], Cuilei Wei[1], Yutao Wei[5], Yan Qi[1], Jianming Hu[1], Weihua Liang[1], Wen Jie Zhang[1], Mei Wan[6], Feng Li[1*]

[1]Department of Pathology and Key Laboratory of Xinjiang Endemic and Ethnic Diseases (Ministry of Education), Shihezi University School of Medicine, Shihezi 832002, Xinjiang, China
[2]Department of Public Health, Medical School, Shihezi University School of Medicine, Shihezi 832002, Xinjiang, China
[3]Department of Clinical Laboratory, First Affiliated Hospital to Shihezi University School of Medicine, Shihezi 832008, Xinjiang, China
[4]Department of Stomatology, First Affiliated Hospital to Shihezi University School of Medicine, Shihezi 832008, Xinjiang, China
[5]Department of Thoracic and Cardiovascular Surgery, First Affiliated Hospital to Shihezi University School of Medicine, Shihezi 832008, Xinjiang, China.
[6]Department of Orthopedic Surgery, Johns Hopkins University School of Medicine, Baltimore, MD 21205, USA

# First author: Dr. Lijuan Pang, M.D, Ph.D E-mail: ocean123456@163.com

[*]Corresponding author: Dr. Feng Li, M.D, Ph.D Department of Pathology and Key Laboratory of Xinjiang Endemic and Ethnic Diseases (Ministry of Education), Shihezi University School of Medicine (E-mail: lifeng7855@126.com)

Supplement Table S2. Snail and Slug protein expression in relation to clinical pathological characteristics in Kazakh ESCC.

Table S2

Clinicopathologica	Snail			z	Slug			z
features	-	+	2+/3+	P	-	+	2+/3+	P
N	22	15	5	-4.562	24	16	2	-4.635
Ca	13	40	35	<**0.001**	23	24	41	<**0.001**
Sex								
Male	5	31	19	-0.114	15	19	21	-0.923
Female	8	9	16	0.909	8	5	20	0.356
Age (y)								
<60	6	22	13	-1.118	11	7	23	-0.81
≥60	7	18	22	0.264	12	17	18	0.408
Tumor invasion								
Superficial layer	5	20	24	-2.094	11	12	26	-1.017
Deep layer	8	20	11	0.036	12	12	15	0.309
LN metastases								
Yes	8	27	22	-0.133	15	14	28	-0.419
No	5	13	13	0.894	8	10	13	0.675
Tumor differentiation								
Well	8	13	4	-3.402	7	9	9	-0.967
Moderate-poor	5	27	31	**0.001**	16	15	32	0.333
Stage								
I + II	9	21	34	-3.399	12	16	36	-3.151
III+ IV	4	19	1	**0.001**	11	8	5	**0.002**

P-values ≤ 0.05 are in bold.
