# Supplementary material for: Membrane type 1-matrix metalloproteinase induces epithelial-to-mesenchymal transition in esophageal squamous cell carcinoma: Observations from clinical and in vitro analyses
Source: Sci Rep. 2016 Feb 26;6:22179. doi: 10.1038/srep22179 (PMC4768157; doi:10.1038/srep22179)
Supplement: Supplement Table S3 [file srep22179-s3.pdf]

Membrane type 1-matrix metalloproteinase induces epithelial-to-mesenchymal transition in esophageal squamous cell carcinoma: Observations from clinical and *in vitro* analyses

Lijuan Pang[1#], Qiuxiang Li[1], Shugang Li[2], Jianwei He[3], Weiwei Cao[1], Jiaojiao Lan[1], Bin Sun[4], Hong Zou[1], Chengyan Wang[1], Ruixue Liu[1], Cuilei Wei[1], Yutao Wei[5], Yan Qi[1], Jianming Hu[1], Weihua Liang[1], Wen Jie Zhang[1], Mei Wan[6], Feng Li[1*]

[1]Department of Pathology and Key Laboratory of Xinjiang Endemic and Ethnic Diseases (Ministry of Education), Shihezi University School of Medicine, Shihezi 832002, Xinjiang, China
[2]Department of Public Health, Medical School, Shihezi University School of Medicine, Shihezi 832002, Xinjiang, China
[3]Department of Clinical Laboratory, First Affiliated Hospital to Shihezi University School of Medicine, Shihezi 832008, Xinjiang, China
[4]Department of Stomatology, First Affiliated Hospital to Shihezi University School of Medicine, Shihezi 832008, Xinjiang, China
[5]Department of Thoracic and Cardiovascular Surgery, First Affiliated Hospital to Shihezi University School of Medicine, Shihezi 832008, Xinjiang, China.
[6]Department of Orthopedic Surgery, Johns Hopkins University School of Medicine, Baltimore, MD 21205, USA

# First author: Dr. Lijuan Pang, M.D, Ph.D E-mail: ocean123456@163.com

[*]**Corresponding author: Dr. Feng Li, M.D, Ph.D** Department of Pathology and Key Laboratory of Xinjiang Endemic and Ethnic Diseases (Ministry of Education), Shihezi University School of Medicine (E-mail: lifeng7855@126.com)

Supplement Table S3. Correlation between the expression of E-CAD protein and Snail, Slug Proteins in ESCC.

Table S3

E-CAD	Snail			Slug		
	-	+	2+/3+	-	+	2+/3+
_	1	2	20	1	1	21
+	4	37	15	14	22	20
2+/3+	8	1	0	8	1	0
	r=-0.648 **P<0.001**			r=-0.594 **P<0.001**		

P-values ≤ 0.05 are in bold.
